# Establishment and validation of a nomogram model for predicting distant metastasis in medullary thyroid carcinoma: An analysis of the SEER database based on the AJCC 8th TNM staging system

**DOI:** 10.3389/fendo.2023.1119656

**Published:** 2023-02-15

**Authors:** Zhufeng Chen, Yaqian Mao, Tingting You, Gang Chen

**Affiliations:** ^1^ Shengli Clinical Medical College, Fujian Medical University, Fuzhou, China; ^2^ Department of Internal Medicine, Fujian Provincial Hospital South Branch, Fuzhou, China; ^3^ Department of Medical Intensive Care Unit, Fujian Provincial Hospital, Fuzhou, China; ^4^ Department of Endocrinology, Fujian Provincial Hospital, Fuzhou, China

**Keywords:** medullary thyroid carcinoma (MTC), distant metastasis, risk factors, nomogram model, cancer-specific survival

## Abstract

**Objective:**

Medullary thyroid carcinoma (MTC) patients with distant metastases frequently present a relatively poor survival prognosis. Our main purpose was developing a nomogram model to predict distant metastases in MTC patients.

**Methods:**

This was a retrospective study based on the Surveillance, Epidemiology, and End Results (SEER) database. Data of 807 MTC patients diagnosed from 2004 to 2015 who undergone total thyroidectomy and neck lymph nodes dissection was included in our study. Independent risk factors were screened by univariate and multivariate logistic regression analysis successively, which were used to develop a nomogram model predicting for distant metastasis risk. Further, the log‐rank test was used to compare the differences of Kaplan-Meier curves of cancer-specific survival (CSS) in different M stage and each independent risk factor groups.

**Results:**

Four clinical parameters including age > 55 years, higher T stage (T3/T4), higher N stage (N1b) and lymph node ratio (LNR) > 0.4 were significant for distant metastases at the time of diagnosis in MTC patients, and were selected to develop a nomogram model. This model had satisfied discrimination with the AUC and C-index of 0.894, and C-index was confirmed to be 0.878 through bootstrapping validation. A decision curve analysis (DCA) was subsequently made to evaluate the feasibility of this nomogram for predicting distant metastasis. In addition, CSS differed by different M stage, T stage, N stage, age and LNR groups.

**Conclusions:**

Age, T stage, N stage and LNR were extracted to develop a nomogram model for predicting the risk of distant metastases in MTC patients. The model is of great significance for clinicians to timely identify patients with high risk of distant metastases and make further clinical decisions.

## Introduction

Medullary thyroid carcinoma (MTC) is a rare neuroendocrine malignant tumor that arises from parafollicular C-cells. About 75% MTC are sporadic and the remaining 25% are hereditary (1, 2). Germline mutations of the *RET* proto-oncogene cause virtually all hereditary MTC, whereas somatic *RET* mutations have been described in approximately 50% sporadic MTC samples (3). Although it accounts for less than 5% of thyroid cancer, it causes approximately 13%-14% of thyroid cancer-related deaths (2–6). Distant metastasis is a poor prognostic factor in MTC patients (5, 7, 8). However, about 10%-15% of MTC patients had distant metastases at the time of initial diagnosis (2, 9, 10). A retrospective study of MTC with distant metastases found that the rate of distant metastases detected postoperative was even higher than that preoperative, and most of included patients (89.1%) had undergone total thyroidectomy and central neck dissection (11). The most common metastatic sites of MTC are the lung, bone and liver (11–16). According to previous studies, the 10-year survival rate of MTC patients is greater than 80% (4, 5, 10, 17). However, in the presence of distant metastases, the 10-year disease-specific survival rate drops to 26-44% (2, 4).

Targeted therapy has brought great hope to patients with advanced progressive MTC over the past decade. Selpercatinib and pralsetinib, two RET-specific inhibitors recently approved by the FDA for the treatment of *RET*-mutant MTC, have been shown to be effective and well tolerated (18, 19). MTC patients with distant metastases are expected to improve survival through targeted therapy. Therefore, it is critical to timely and effective identification of MTC patients at high risk of distant metastases.

According to American Thyroid Association Guidelines, contrast-enhanced CT of the neck and chest, three-phase contrast-enhanced CT or contrast-enhanced MRI of the liver, and axial MRI and bone scintigraphy are recommended in MTC patients with suspected distant metastases (3). However, the sensitivity of these imaging techniques in localizing metastatic disease is below 80% (3), and these systemic imaging techniques are expensive to repeat during postoperative monitoring.

Currently, there are very rare models for predicting distant metastases of MTC patients. Therefore, it is of great clinical significance and economic benefit to construct a simple and convenient clinical model for predicting the distant metastasis in MTC. We designed a retrospective study based on the SEER database, which mainly aimed at constructing and validating a nomogram to predict distant metastases for MTC patients.

## Materials and methods

### Patients and data collection

The data we analyzed were generated from the SEER database using the SEER∗Stat software (version 8.3.9.2; National Cancer Institute, USA). The SEER database was last updated in November 2020, and the last follow-up date for the updated data was late 2018.

Inclusion criteria were the following: (1) Patients diagnosed with primary cancer from 2004 to 2015; (2) According to the International Classification of Diseases (ICD) for Oncology-3, patients histological codes were codes 8345/3 and 8510/3, Primary site code C73.9; (3) All included patients were undergone total thyroidectomy and neck lymph nodes dissection. Data such as year of diagnosis, sex, age of diagnosis, race (white, black, other), marital status at diagnosis (married, single, divorced/separated/widowed), primary site, histologic type, tumor-node-metastasis (TNM) stage at the time of diagnosis, tumor size, tumor extension, CS site-specific factor 1 (multifocality), surgery of primary site, scope of regional lymph node surgery, regional nodes examined, regional nodes positive, survival months, cause of death, and vital survival status were collected from the SEER database. Exclusion criteria included: (1) Patients with more than one kind of primary malignant cancer; (2) Patients aged < 18 years or > 90 years; (3) Patients with survival time less than 1 month; (4) Patients without regional lymph nodes examined; (5) Patients with unknown or missing clinical information. Finally, data of 807 patients were selected from the SEER database based on the inclusion and exclusion criteria.

**Figure 1:**
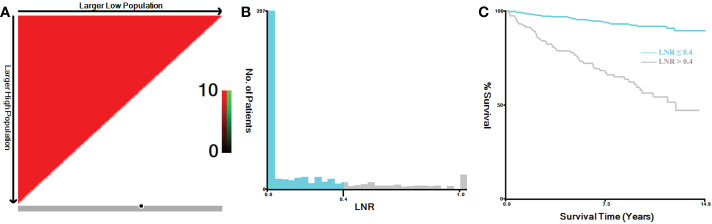
X-tile analysis of MTC patients for the relationship between LNR and the CSS. The training plots are shown in panel **(A)**, with matched validation sets shown in **(B, C)**. The optimal cut-point highlighted by the black circle in panel **(A)** is shown on a histogram of the entire cohort **(B)** and a Kaplan-Meier curve **(C)** (*p* < 0.001). MTC, Medullary thyroid carcinoma; LNR, lymph node ratio.

**Figure 2:**
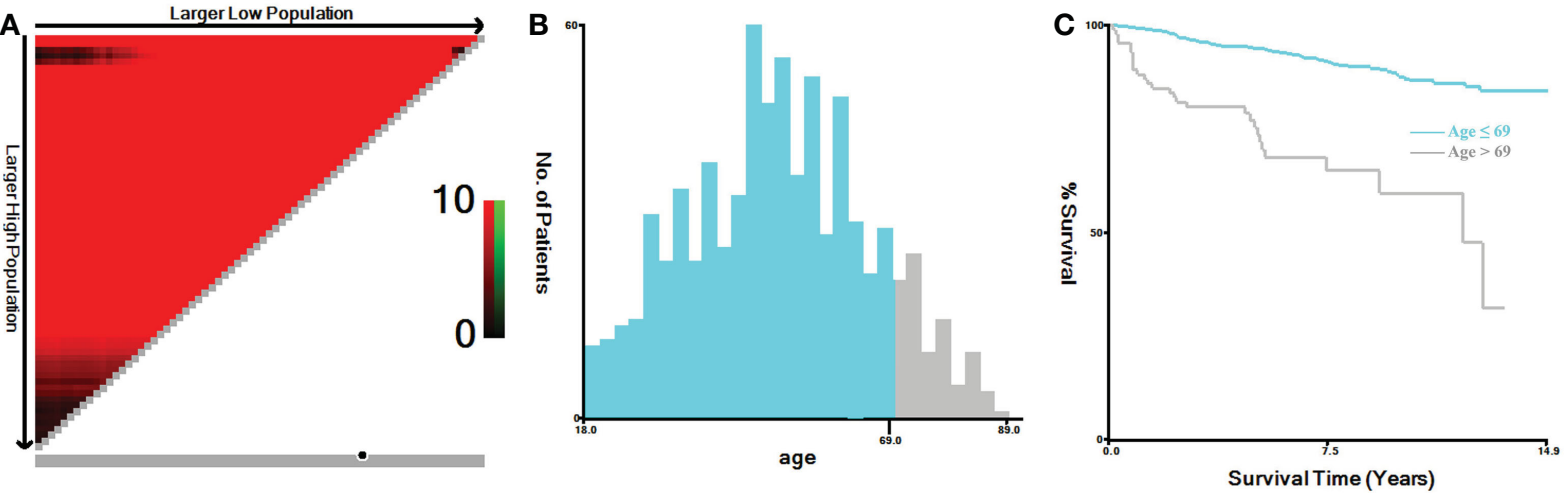
X-tile analysis of MTC patients for the relationship between age and the CSS. The training plots are shown in panel **(A)**, with matched validation sets shown in **(B, C)**. The optimal cut-point highlighted by the black circle in panel **(A)** is shown on a histogram of the entire cohort **(B)** and a Kaplan-Meier curve **(C)** (*p* < 0.001). MTC, Medullary thyroid carcinoma.

In this study, TNM stage at the time of diagnosis for all cases were converted into the 8th Edition of the American Joint Committee on Cancer (AJCC) TNM staging system (20). Primary tumor (T stage) was categorized as T1, T2, T3 and T4. Regional lymph node (N stage) was categorized as N0, N1a and N1b. Distant metastasis (M stage) was categorized as M0 and M1. Tumor expansion classification includes with or without gross extra-thyroidal extension (ETE). Based on the regional nodes examined and regional nodes positive of each case, we calculated the lymph node ratio (LNR), which represented the proportion of metastatic lymph nodes resected. The continuous variables of the LNR and age were analyzed by X-tile software, both of which were grouped as binary variables (21). The best cut-off value for LNR was 0.4 (**Figures 1A-C**), and for age was 69 years (**Figures 2A-C**). In addition, the study predicting MTC overall survival by A. Kotwal et al. identified 55 years as cut point (10).

### Statistical analysis

Statistical analyses were conducted using the SPSS software (version 23.0; IBM Corporation) and R (Version 4.1.3; https://cloud.r-project.org/) software. Continuous variables were presented as mean ± standard deviation or median with interquartile range, as appropriate. Categorical variables were presented as number and percentage. The χ^2^ test or Fisher’s exact test was used for categorical variables. Univariate logistic regression analysis was used to screen potential risk factors for distant metastasis. Based on Odds ratios (ORs) for age categorical variables, we included age categorical variable (≤ 55 and > 55) in the multivariate logistic regression analysis. Subsequently, independent risk factors were further screened by multivariate logistic regression analysis (Method: Forward: LR). ORs and 95% confidence intervals (CIs) were also reported for each risk factor.

All independent risk factors were applied to develop a nomogram model predicting distant metastasis risk. The Harrell’s concordance index (C-index), receiver operating characteristic curve (ROC) and the calibration curve of MTC patients were formulated to evaluate the exact predictive performance of the nomogram model. The Bootstrap method (1,000 bootstrap resamples) was used to conduct internal verification of the nomogram model. Decision curve analysis (DCA) was also performed to determine the clinical utility of the predictive model.

The log‐rank test was used to compare the differences of Kaplan-Meier curves of cancer-specific survival (CSS) in different groups, and cumulative CSS rates were calculated. All tests were 2-sided, and a *p* value < 0.05 was considered statistically significant.

### Ethical approval

The SEER database is public, does not contain any personally identifiable information, and therefore does not require ethical approval. Our request for access to SEER Data has been approved from the National Cancer Institute, USA (reference number 23459-Nov2020).

## Results

### Clinical parameters of MTC patients

The mean age of all included MTC patients was 51.29 ± 15.46 years, of whom 470 (58.2%) were female, 337 (41.8%) were male; 332 (41.1%) were in the T1 stage, 249 (30.9%) were in the T2 stage, 153 (19.0%) were in the T3 stage, and 73 (9.0%) were in the T4 stage; 407 (50.4%) had no regional lymph node metastases, 171 (21.2%) had central regional lymph node metastases (N1a), 229 (28.4%) had lateral regional lymph node metastases (N1b); 754 (93.4%) had no distant metastases (M0), 53 (6.6%) had distant metastases (M1); Tumor size 368 (45.6%) were ≤ 20mm, 299 (37.1%) were 21- 40mm, and 140 (17.3%) were > 40mm; 681 (84.4%) were without gross ETE, 126 (15.6%) were with gross ETE; 553 (68.5%) were unifocal tumor, 254 (31.5%) were multifocal tumor; the median regional nodes examined was 14.00 (5.00, 34.00), and the median regional nodes positive was 0 (0, 7.00). The median follow-up time for all MTC patients was 86.00 (53.00, 120.00) months. Their basic clinical parameters of the MTC patients were summarized in **Table 1**.

**Table 1:** Comparison of main clinical parameters of the MTC patients with and without distant metastases.

	Total (n=807)	With DM (n1 = 53)	Without DM (n2 = 754)	P value
*Age (years)*				< 0.001
≤ 55	497(61.6%)	20(37.7%)	477(63.3%)	
> 55	310(38.4%)	33(62.3%)	277(36.7%)	
≤ 69	694(86.0%)	40(75.5%)	654(86.7%)	0.022
> 69	113(14.0%)	13(24.5%)	100(13.3%)	
*Sex*				0.001
Female	470(58.2%)	19(35.8%)	451(59.8%)	
Male	337(41.8%)	34(64.2%)	303(40.2%)	
*Marital status*				0.471
Married	544(67.4%)	33(62.3%)	511(67.8%)	
Single	160(19.8%)	14(26.4%)	146(19.4%)	
Divorced/Separated/Widowed	103(12.8%)	6(11.3%)	97(12.9%)	
*Race*				0.580
White	682(84.5%)	43(81.1%)	639(84.7%)	
Black	67(8.3%)	6(11.3%)	61(8.1%)	
Other	58(7.2%)	4(7.5%)	54(7.2%)	
*T Stage*				< 0.001
T1	332(41.1%)	4(7.5%)	328(43.5%)	
T2	249(30.9%)	7(13.2%)	242(32.1%)	
T3	153(19.0%)	22(41.5%)	131(17.4%)	
T4	73(9.0%)	20(37.7%)	53(7.0%)	
*N Stage*				< 0.001
N0	407(50.4%)	3(5.7%)	404(53.6%)	
N1a	171(21.2%)	12(22.6%)	159(21.1%)	
N1b	229(28.4%)	38(71.7%)	191(25.3%)	
*Tumor size (mm)*				< 0.001
≤ 20	368(45.6%)	8(15.1%)	360(47.7%)	
21- 40	299(37.1%)	18(34.0%)	281(37.3%)	
> 40	140(17.3%)	27(50.9%)	113(15.0%)	
*Gross ETE*				< 0.001
No	681(84.4%)	23(43.4%)	658(87.3%)	
Yes	126(15.6%)	30(56.6%)	96(12.7%)	
*Multifocality*				0.053
Unifocal	553(68.5%)	30(56.6%)	523(69.4%)	
Multifocal	254(31.5%)	23(43.4%)	231(30.6%)	
*LNR*				< 0.001
≤ 0.4	639(79.2%)	19(35.8%)	620(82.2%)	
> 0.4	168(20.8%)	34(64.2%)	134(17.8%)	

MTC, Medullary thyroid carcinoma; DM, distant metastasis; Gross ETE, Gross extra-thyroidal extension; LNR, lymph node ratio.

### Selection of risk factors for distant metastasis

Analyses were performed using univariate and multivariate logistic regression to identify clinical parameters affecting distant metastasis at the time of diagnosis. In univariate logistic regression analyses, age > 55 years, age > 69 years, male sex, higher T stage (T3/T4), higher N stage (N1a/N1b), large tumor size (21-40/> 40), gross ETE and LNR > 0.4 were significant (*p* < 0.05) factors (**Table 2**). According to the calculated OR values, we selected 55 instead of 69 years as the preferred cut-off value for age (OR = 2.841, 95%CI: 1.599-5.048, *p* < 0.001; OR = 2.125, 95%CI: 1.098-4.113, *p* = 0.025, respectively). In the multivariate logistic regression analysis (Method: Forward: LR), age > 55 years (OR = 3.226, 95%CI: 1.694-6.146, *p* < 0.001), higher T stage (T3: OR = 5.280, 95%CI: 1.678-16.612, *p* = 0.004; T4: OR = 8.581, 95%CI: 2.615-28.151, *p* < 0.001), higher N stage (N1b: OR = 7.244, 95%CI: 1.972-26.619, *p* = 0.003) and LNR > 0.4 (OR = 3.081, 95%CI: 1.526-6.222, *p* = 0.002) all entered the equation, so were identified as an independent risk factors for distant metastasis, while sex, tumor size and gross ETE did not enter the equation (**Table 2**).

**Table 2:** Univariate and multivariate logistic regression analysis of distant metastases in the MTC patients.

	Univariate analysis			Multivariate analysis		
	OR	95% CI	*P* value	OR	95% CI	*p* value
*Age (years)*						
≤ 55	Ref			Ref		
> 55	2.841	1.599-5.048	< 0.001	3.226	1.694-6.146	< 0.001
≤ 69	Ref					
> 69	2.125	1.098-4.113	0.025			
*Sex*						
Female	Ref					
Male	2.664	1.491-4.757	0.001	—	—	—
*Marital status*						
Married	Ref					
Single	1.485	0.774-2.849	0.234	—	—	—
Divorced/Separated/Widowed	0.958	0.391-2.348	0.925	—	—	—
*Race*						
White	Ref					
Black	1.462	0.598-3.572	0.405	—	—	—
Other	1.101	0.381-3.182	0.859	—	—	—
*T Stage*						
T1	Ref			Ref		
T2	2.372	0.687-8.193	0.172	1.808	0.504-6.487	0.364
T3	13.771	4.656-40.732	< 0.001	5.280	1.678-16.612	0.004
T4	30.943	10.177-94.085	< 0.001	8.581	2.615-28.151	< 0.001
N Stage						
N0	Ref			Ref		
N1a	10.164	2.830-36.496	< 0.001	2.677	0.640-11.202	0.178
N1b	26.792	8.168-87.887	< 0.001	7.244	1.972-26.619	0.003
*Tumor size (mm)*						
≤ 20	Ref					
21- 40	2.883	1.235-6.726	0.014	—	—	—
> 40	10.752	4.751-24.334	< 0.001	—	—	—
*Gross ETE*						
No	Ref					
Yes	8.940	4.986-16.030	< 0.001	—	—	—
*Multifocality*						
Unifocal	Ref					
Multifocal	1.736	0.987-3.054	0.056	—	—	—
*LNR*						
≤ 0.4	Ref			Ref		
> 0.4	8.280	4.582-14.961	< 0.001	3.081	1.526-6.222	0.002

MTC, Medullary thyroid carcinoma; Gross ETE, Gross extra-thyroidal extension; LNR, lymph node ratio.

### Development and validation of the nomogram for distant metastasis

**Figure 3:**
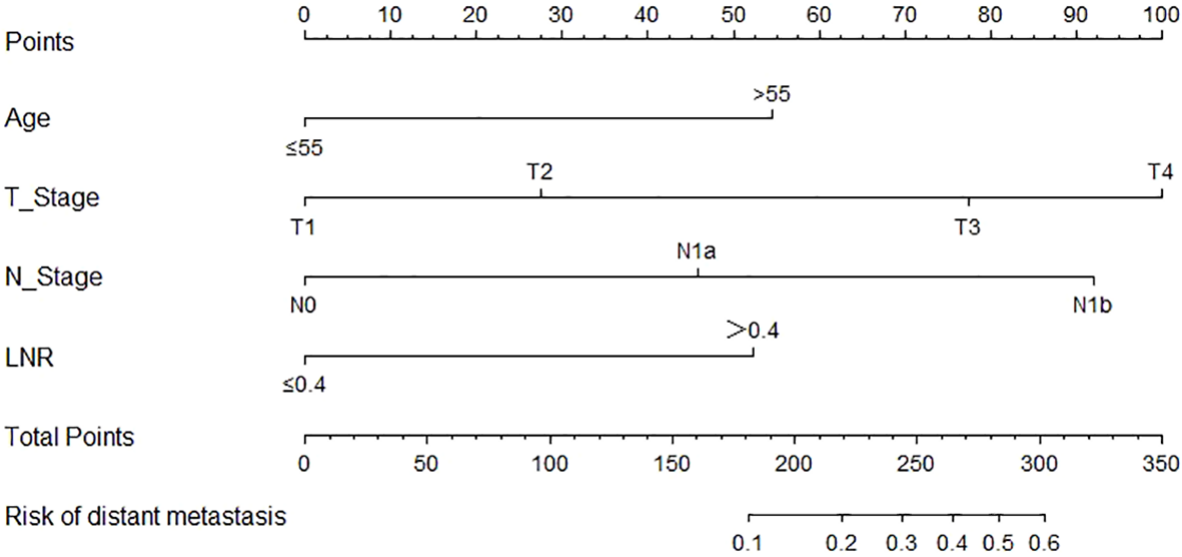
Nomogram for predicting distant metastasis in MTC. MTC, Medullary thyroid carcinoma; LNR, lymph node ratio.

**Figure 4:**
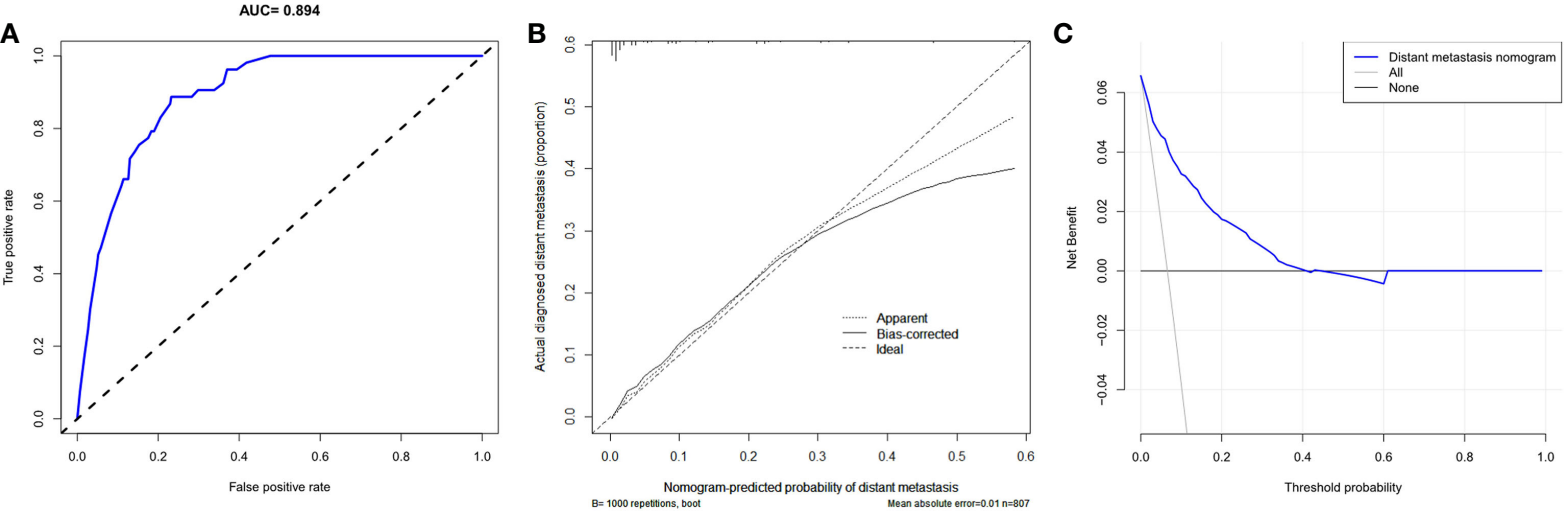
Predictive performance of a nomogram model for predicting distant metastasis in MTC. **(A)** ROC curve analysis to predict distant metastasis in MTC; **(B)** Calibration curve of the nomogram for predicting distant metastasis in MTC; **(C)** Decision curve analysis for distant metastasis in MTC. ROC, Receiver operating characteristic curve; MTC, Medullary thyroid carcinoma.

Based on the result of multivariate logistic regression, four variables including age, T stage, N stage and LNR were finally extracted to build the nomogram model for predicting the risk of distant metastasis in MTC (**Figure 3**). According to the plotted ROC curve (**Figure 4A**), the AUC of the nomogram was 0.894. The C-index for the nomogram model was 0.894 (95%CI: 0.862-0.926), and was confirmed to be 0.878 through bootstrapping validation, which showed that the nomogram model had a high accuracy prediction. The calibration curve demonstrated good consistency between the predicted result and the actual distant metastasis state of the MTC (**Figure 4B**).

### Decision curve analysis for clinical utility

As can be seen in the DCA for the distant metastasis nomogram (**Figure 4C**), the net benefit (NB) of the decision curve of the model is higher than that of the two invalid lines within the range of threshold probability between 1%-40%.

### Cancer-specific survival for different M stage, T stage, N stage, age, and LNR

**Figure 5:**
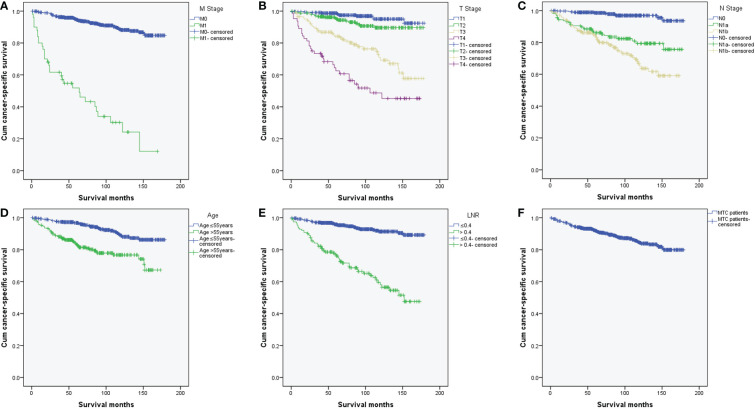
Kaplan-Meier curves of cancer-specific survival in MTC patients. **(A)** K-M curves of CSS differed by different M stage (*p* < 0.001); **(B)** K-M curves of CSS differed by different T stage (*p* < 0.001); **(C)** K-M curves of CSS differed by different N stage (*p* < 0.001); **(D)** K-M curves of CSS differed by different age (*p* < 0.001); **(E)** K-M curves of CSS differed by different LNR (*p* < 0.001); **(F)** K-M curve of CSS for all MTC patients. MTC, Medullary thyroid carcinoma; K-M, Kaplan-Meier; CSS, cancer-specific survival; LNR, lymph node ratio.

We further compared the differences of Kaplan-Meier curves of CSS in different groups. CSS differed by different M stage, T stage, N stage, age and LNR groups (**Figures 5A-F**). Overall, the CSS rates of MTC patients with distant metastases were significantly lower than those without distant metastases (χ^2^ = 220.740, *p* < 0.001) (**Figure 5A**). The 3-, and 5-year cumulative CSS of MTC patients without distant metastases were 96.3% and 94.7% while they were reduced to 61.6% and 51.9% with distant metastases. MTC patients with higher T stage, higher N stage, age > 55 years and LNR > 0.4 had lower CSS (χ^2^ = 131.690, *p* < 0.001; χ^2^ = 76.487, *p* < 0.001; χ^2^ = 32.703, *p* < 0.001; χ^2^ = 103.493, *p* < 0.001, respectively) (**Figures 5B-E**).

## Discussion

MTC is a rare carcinoma which is more aggressive than differentiated thyroid carcinoma (DTC). MTC cells do not concentrate radioactive iodine and are not sensitive to thyrotropin-suppressive therapy, which is different from DTC. Although the proportion of MTC in thyroid cancer had declined in recent years, it caused a disproportionately high rate of thyroid-related death (2–6). The survival rate for the MTC is significantly lower than that of DTC (10, 17). According to our results, 6.6% (53/807) of MTC patients had distant metastases. For MTC patients with distant metastases 3- and 5-year cumulative CSS rates were 61.6% and 51.9%. 5-year CSS rate was close to the results of two studies of SEER databases (2, 4).

Several studies have already identified survival prognostic factors for MTC, including age, primary tumor size, initial stage, extrathyroidal extension, lymph node metastasis and initial distant metastasis (2, 3, 5, 8, 10, 11). However, distant metastasis is the strongest predictor of OS and progression-free survival (8). The most common metastatic sites of MTC are the lung, bone and liver (11–16). Lung metastases generally present as multiple micronodular in most patients (11). For bone metastases, the lesions are mostly multifocal and preferentially occur in the spine, pelvis and ribs, and the most common morphologies of bone metastases are osteolytic and osteogenic (22, 23). Moreover, liver metastases are often multiple, and disseminated throughout the parenchyma (24, 25). The most common causes of death from distant metastases are complications from the progression of distant metastases, chemotherapy-related complications, and airway obstruction from tracheal invasion (11).

The recent application of RET-specific inhibitors (selpercatinib and pralsetinib) has provided an effective and promising option for systemic treatment in *RET*-mutant MTC patients with metastatic and progressive diseases (18, 19). However, the high cost of imaging techniques screening for distant metastases reduces the initiative for monitoring, and these techniques still have the possibility of false negatives, which may delay the timing of treatment in patients with advanced MTC. It is of great clinical significance to evaluate the independent risk factors for distant metastasis and further establish a clinical prediction model in MTC.

According to the 8th edition of the AJCC staging system, the TNM classification lacks important prognostic factors such as gradations of age in patients with MTC (20). In addition, the lymph node metastases classifications for MTC in this staging system are just according to the location of nodes, regardless of the number or the rate of lymph nodes metastases (5, 26–29).

Increased age was associated with higher disease specific mortality and worse survival in MTC patients (2, 10, 30–32). The cut-off value analyzed by X-tile software for the relationship between age and the CSS was 69 years (**Figures 2A-C**) (21). While, referring to previous study by A. Kotwal et al., the cut-off value for age was 55 years (10). In univariate logistic regression analyses, age > 55 and > 69 years were all statistically significant for distant metastasis. According to the calculated OR values, we selected 55 years as the preferred cut-off value for age. Age > 55 years could independently predict distant metastasis in MTC.

In our study, the optimal cut-off value for the relationship between LNR and the CSS was 0.4. Previous studies found that higher metastatic lymph node ratio predict poorer survival in MTC (5, 28, 29). The cut-off values for the metastatic lymph node ratio previously selected were 0.1 and 0.5 (28, 33). Different from previous studies, our study was based on all adult MTC patients who underwent total thyroidectomy and neck lymph nodes dissection. Therefore, 0.4, as our cut-off value, is more clinically applicable than previous studies. In addition to predicting survival, the lymph node ratio can potentially predict recurrence and distant metastases in MTC (10, 34). We came to a similar conclusion, LNR > 0.4 was an independent significant predictor of distant metastasis.

Previous studies had proved the value of T stage in predicting survival in MTC (28, 32, 35), but there were few studies for predicting distant metastasis. Our study also confirmed that higher T stage (T3/T4) was an independent risk factor for distant metastasis. N stage could not only predict survival (27, 28, 32), but also for distant metastasis (10, 15). Lateral regional lymph node metastasis (N1b) was an independent predictor for distant metastasis, which was different from central regional lymph node metastasis (N1a) (10).

Univariate logistic analysis in our study showed that male sex and Tumor sizewere potential risk factors for distant metastases in MTC, but they lost significance in the subsequent multivariate logistic analysis, which was similar to the finding of A. Kotwal et al. (10). Some studies suggested that gross ETE could independently predict distant metastasis (10, 26), but it was not an independent predictor of distant metastasis in our multivariate logistic analysis.

We developed the nomogram for distant metastasis based on the four independent predictors mentioned above, including age, T stage, N stage and LNR. To our knowledge, this is a very rare nomogram with good predictive performances to predicted distant metastases of MTC patients who undergone total thyroidectomy and neck lymph nodes dissection. In this model, the C-index and AUC values of the nomogram prediction model were both 0.894, and the C-index value of bootstrapping validation was 0.878, indicating that the model had good predictive ability. The calibration curve suggested that the actual probability of distant metastasis corresponded closely with the predicted probability of distant metastasis in MTC.

The predictive model can help clinicians to screen patients at high risk of distant metastases in MTC. Through the implementation of close postoperative monitoring of these people, timely treatment if necessary, and ultimately improve the poor prognosis of these patients.

The limitation of our study was its retrospective design, small sample size and lacking external validation. Due to the limitations of the SEER database, there was a lack of evaluation of genetic status, calcitonin and carcinoembryonic antigen in our study. Despite these limitations, we verified that age > 55 years, higher T stage (T3/T4), higher N stage (N1b) and lymph node ratio (LNR) > 0.4 were significant predictors of distant metastasis in MTC. We further established a nomogram model for predicting distant metastases in MTC patients. For these MTC patients at high risk of distant metastases, we recommend close monitoring of whole-body imaging procedures after surgery.

## Conclusion

Overall, the survival prognosis of MTC patient with distant metastasis is poor. Based on the SEER database, we found that age > 55 years, higher T stage (T3/T4), higher N stage (N1b) and lymph node ratio (LNR) > 0.4 could independently predict distant metastases in MTC patients. More importantly, we had successfully developed a visual nomogram model to predict distant metastases in MTC patients who undergone total thyroidectomy and neck lymph nodes dissection. The model is of great significance for clinicians to timely identify patients with high risk of distant metastases and make further clinical decisions.

## Data availability statement

The dataset presented in this study can be found in here: https://seer.cancer.gov. Further inquiries can be directed to the corresponding author.

## Author contributions

ZC, YM, TY, and GC contributed to this study. ZC and GC contributed to the conception and design of this study. ZC collected data. YM and TY performed the statistical analysis. ZC, YM, and TY drafted and wrote the manuscript. GC supervised the entire study. All authors contributed to the article and approved the submitted version.
